# Complete Genome Sequence of *Pseudomonas brassicacearum* LBUM300, a Disease-Suppressive Bacterium with Antagonistic Activity toward Fungal, Oomycete, and Bacterial Plant Pathogens

**DOI:** 10.1128/genomeA.01623-15

**Published:** 2016-01-28

**Authors:** Amy Novinscak, Vijay J. Gadkar, David L. Joly, Martin Filion

**Affiliations:** Department of Biology, Université de Moncton, Moncton, New Brunswick, Canada

## Abstract

*Pseudomonas brassicacearum* LBUM300, a plant rhizosphere-inhabiting bacterium, produces 2,4-diacetylphloroglucinol and hydrogen cyanide and has shown antagonistic activity against the plant pathogens *Verticillium dahliae*, *Phytophthora cactorum*, and *Clavibacter michiganensis* subsp. *michiganensis*. Here, we report the complete genome sequence of *P. brassicacearum* LBUM300.

## GENOME ANNOUNCEMENT

*Pseudomonas brassicacearum* LBUM300 is a strawberry (*Fragaria × ananassa*) rhizosphere isolate that has demonstrated *in vitro* antagonistic activity against the fungal and oomycete plant pathogens *Verticillium dahliae* and *Phytophthora cactorum* (1). It has also been shown to be capable of reducing tomato plant symptoms of bacterial canker caused by *Clavibacter michiganensis* subsp. *michiganensis* (2). The antagonistic activity of *P. brassicacearum* LBUM300 has been associated with its ability to produce 2,4-diacetylphloroglucinol (DAGP) and hydrogen cyanide (HCN) (1, 2). The genome of *P. brassicacearum* LBUM300 was analyzed to better understand its potential as a biocontrol agent.

Genomic DNA was extracted using the UltraClean microbial DNA isolation kit (Mo Bio, Carlsbad, CA, USA) and purified using Agencourt AMPure XP beads (Beckman Coulter, Mississauga, Canada), according to the manufacturer’s instructions. The genome of *P. brassicacearum* LBUM300 was sequenced using PacBio single-molecule real-time (SMRT) sequencing technology (McGill University and Génome Québec Innovation Centre, Montreal, Canada). A total of 192,454 raw subreads with an average length of 5,822 bp (144× coverage) were generated using 3 SMRT cells in a PacBio RSII sequencer. Genome assembly was performed using the Hierarchical Genome Assembly Process (HGAP) (3), generating a circular 6,976,764-bp chromosome with an overall G+C content of 60.75%. No functional plasmid was detected. Genome annotation was performed using the Rapid Annotations using Subsystems Technology (RAST) annotation server (4) and identified 6,125 predicted protein-coding sequences, 16 rRNA operons, and 67 tRNA loci.

Ten core housekeeping genes (*acsA*, *aroE*, *dnaE*, *guaA*, *gyrB*, *mutL*, *ppsA*, *pyrC*, *recA*, and *rpoB* [5]) from 48 closely related *Pseudomonas* spp. were retrieved from DDBJ/ENA/GenBank and compared based on concatenated alignments using the CLC Genomics Workbench software version 8.0 (CLC bio, Boston, MA). The resulting maximum-likelihood phylogenetic tree indicated that *P. brassicacearum* LBUM300 belongs to *Pseudomonas* sp. subclade 2, according to Loper et al. (5). Within this subclade, *P. brassicacearum* subsp. *brassicacearum* NFM421 was identified as the closest relative of *P. brassicacearum* LBUM300. Genome analysis of *P. brassicacearum* LBUM300 showed that this strain possesses only one copy of the *phlABCD* and *hcnABC* operons responsible for the biosynthesis of DAPG and HCN, respectively. Furthermore, genes involved in 1-aminocyclopropane-1-carboxylate deaminase (*acdS*) and pyrroloquinoline-quinone (*pqqABCDEF*) production, which might be associated with plant growth-promoting activity, were detected. *P. brassicacearum* LBUM300 also possesses several secretory systems, including type I, II, and III secretion systems, and the biosynthetic and membrane receptor genes associated with pyoverdine production and uptake**.**

### Nucleotide sequence accession number.

This complete genome project has been deposited in DDBJ/EMBL/GenBank under the accession number CP012680. The version described in this paper is the first version.
